# Advances in the Studies of Ginkgo Biloba Leaves Extract on Aging-Related Diseases

**DOI:** 10.14336/AD.2017.0615

**Published:** 2017-12-01

**Authors:** Wei Zuo, Feng Yan, Bo Zhang, Jiantao Li, Dan Mei

**Affiliations:** ^1^Peking Union Medical College Hospital, Chinese Academy of Medical Sciences & Peking Union Medical College, Beijing, China; ^2^Cerebrovascular Diseases Research Institute, Xuanwu Hospital of Capital Medical University, Beijing, China; ^3^Department of Neurobiology, Capital Medical University, Beijing, China

**Keywords:** Ginkgo biloba leaves extract, degenerative disorders, antioxidant, neuron protection, anticancer, cardiovascular

## Abstract

The prevalence of degenerative disorders in public health has promoted in-depth investigations of the underlying pathogenesis and the development of new treatment drugs. Ginkgo biloba leaves extract (EGb) is obtained from Ginkgo biloba leaves and has been used for thousands of years. In recent decades, both basic and clinical studies have established the effects of EGb. It is widely used in various degenerative diseases such as cerebrovascular disease, Alzheimer’s disease, macroangiopathy and more. Here, we reviewed several pharmacological mechanisms of EGb, including its antioxidant properties, prevention of mitochondrial dysfunctions, and effect on apoptosis. We also described some clinical applications of EGb, such as its effect on neuro and cardiovascular protection, and anticancer properties. The above biological functions of EGb are mainly focused on aging-related disorders, but its effect on other diseases remains unclear. Thus, through this review, we aim to encourage further studies on EGb and discover more potential applications

Advancing age is a complex and irreversible process induced by many factors. It is a major risk factor for progression of various diseases and can lead to a decrease in physiological capacity, functional tissue impairment and mortality. There has been much research focusing on the molecular mechanisms of aging, yet the mechanisms are still not thoroughly understood.

Ginkgo biloba is a traditional Chinese medicine that has been used in many different disorders for many centuries. Ginkgo biloba extract (EGb) is derived from the leaves of the maidenhair tree. Its standardized special extract, EGb761, contains different kinds of flavone glycosides and terpenoides. Chemical structures for the constituents of EGb 761 are shown in Fig. 1, and German commission E evaluated it as follows: flavone glycosides 22%-27%, terpene lactones including ginkgolides A, B and C (2.8%-3.4%) and bilobalide (2.6%-3.2%) and less than 5ppm ginkgolic acids, constituents of known allergic and cytotoxic potency [1]. However, EGb 761 used in most clinical trials has been standardized. Interestingly, commission E monograph specifies a range of terpene lactones (5%-7%), but not a limit [2]. Recently, researchers have focused more on EGb instead of Ginkgo biloba itself, EGb is now widely used in research and clinical trials on age-associated diseases including brain dysfunction [3], cardiovascular system diseases [4], carcinogen metabolism [5] and some sensorial tissues diseases [6]

**Figure 1.**
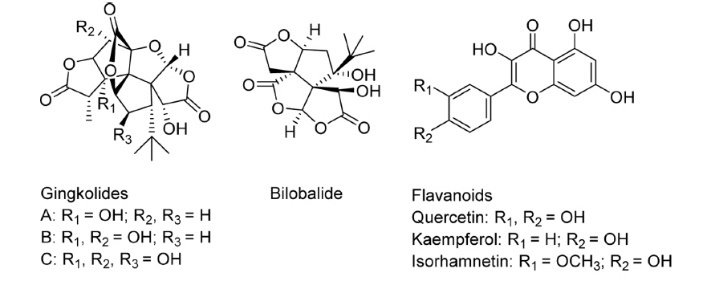
Structures of EGb 761 constituents.

Despite the wide use of EGb in research, there is still debate about its mechanisms, clinical efficacy and clinical application. In this paper, we reviewed the various pharmacological actions of EGb in aging disorders, including its antioxidant properties, and the effect on anti-mitochondrial dysfunction, apoptosis and anti-tumor activity, as well as review relevant clinical trials.

## 1. Pharmacological actions of EGb

### 1.1 Antioxidant properties of EGb

**Figure 2.**
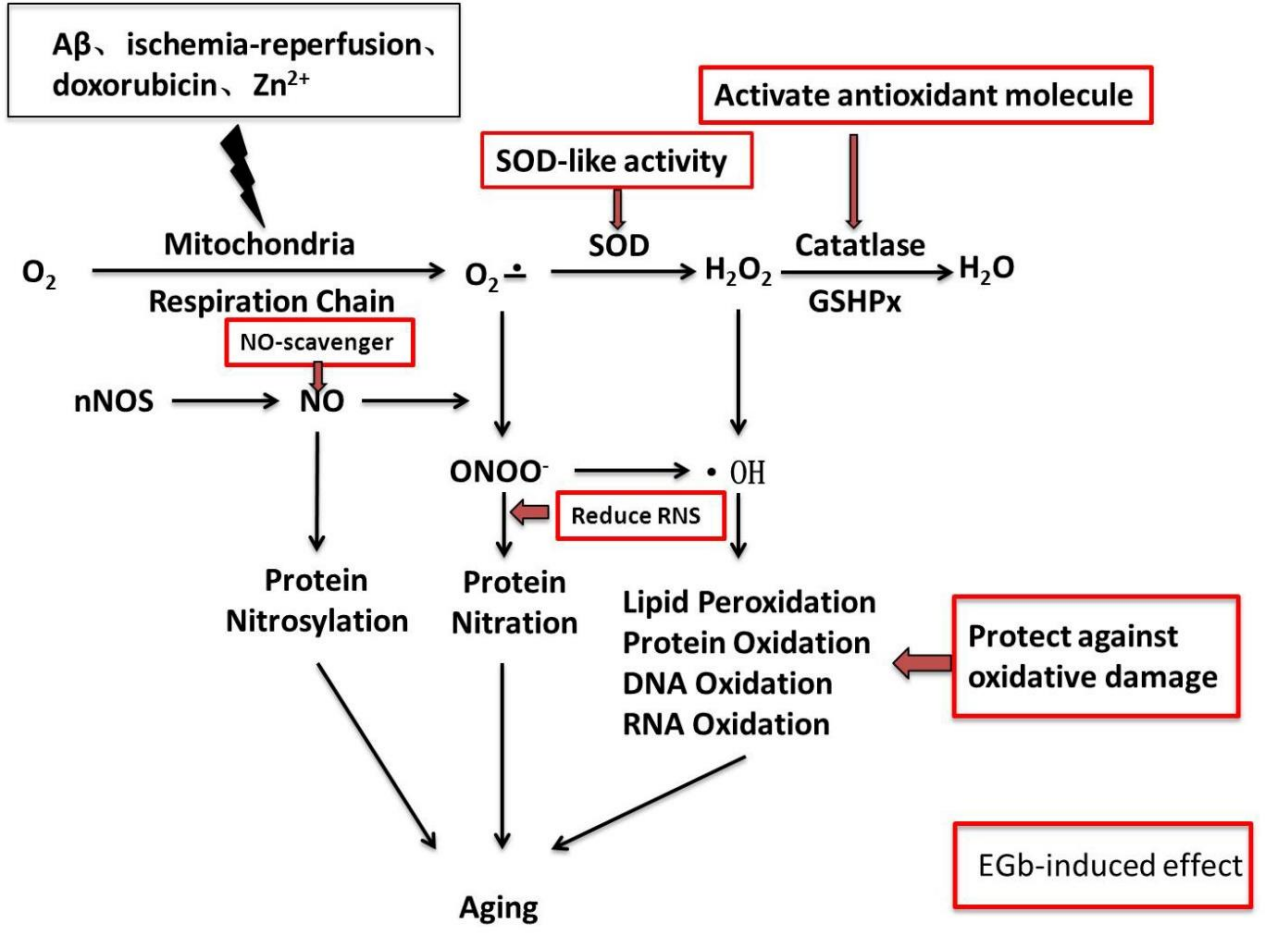
Main resources and pathways for oxidant generateon in aging O^2-^ and NO are produced in various conditions. NOS induces protein nitrosylation as well as the generation of ONOO^-^ by reacting with O^2-^. SOD detoxifies O^2-^ to H_2_O_2_, which is converted to H2O by catalase or GSHPx. OH that is generated from H_2_O_2_ leads cell injury by oxidized lipid, protein, DNA and RNA. EGb could exert an antioxidant effect by scavenging free radical, regulating oxidase and antioxidation enzyme, inhibiting lipid/Protein/DNA/RNA peroxidation and protecting mitochondrial respiratory chain.

During the aging process, tissues suffer DNA oxidative damage, protein oxidative injury, lipid peroxidation, etc (Fig. 2). Subsequently, neurological function, sensorial tissues and cardiovascular system can all be impaired [7]. These impairments significantly contribute to the progression of degenerative diseases [8].

#### Scavenging free radicals

Free radicals include the oxygen free radical, hydroxyl radical, and the singlet oxygen. In 1997, Pietri and colleagues [9] found that EGb 761 could inhibit the generation of free radicals in cardiovascular ischemia, which could be attributed to its SOD-like activity that scavenges hydroxyl radicals[10, 11]. Upon studying EGb’s stoichiometry and kinetics, EGb was found to scavenge the superoxide radical, diphenyl-p-picrylhydral radical and hydroxyl radical, which was dependent on the amount of EGb present [12]. A large number of subsequent studies have focused on the antioxidant abilities of EGb *in vivo* and *in vitro*. Song [13] found that EGb 761 could inhibit ROS accumulation induced by cisplatin in the porcine kidney proximal tubular epithelial (LLC-PK1) cell line. Kaur and colleagues [14] reported that Ginkgo B, a compound from EGb, could reduce the level of ROS/RNS induced by prooxidant Aβ25-35 peptide in human neuroblastoma IMR-32 and SHSY5Y cells. *In vivo*, Kwon, *et al* [15] demonstrated that EGb 761(48 mg/kg) could directly remove ROS in ischemia-reperfusion gerbils.

#### Regulation of oxidativestress

Zhou and colleagues [16] examined the levels of glutathione, malondialdehyde, superoxide dismutase and nitric oxide in ischemia-reperfusion brain tissues. Using a middle cerebral artery occlusion model, they found that the levels of glutathione and superoxide dismutase decreased with age, while pretreatment with EGb 761 could increase their levels in the counterpart MCAO model. On the other hand, the levels of malondialdehyde and nitric oxide increased with age, and pretreatment with EGb761 could suppress them. EGb 761 could be protective against ischemic injury by regulating oxidative stress. Aydin D induced oxidation in rat brains by administering cisplatin and found that EGb 761 could decrease the level of NO and GSH in brain tissue, thereby reducing oxidative stress[17]. Similarly, EGb 761 possessed the ability to reverse the decrease in GSH and GSH-peroxidase levels induced by intermittent hypoxia in rats [18]. Zinc played an important role in the brain, as it was proposed to act as an enzyme capable of regulating ROS levels [19]. Kwon et al [20] reported that EGb could attenuate zinc-induced increases in intracellular ROS levels in rat primary cortical neurons. Yeh and colleagues [21] demonstrated that EGb pretreatment could relieve doxorubicin-induced reduction of superoxide dismutase and activate the antioxidant molecules such as glutathione peroxidase and glutathione in the rat testes.

#### Anti-lipid peroxidation

Polyunsaturated fatty acids (PUFAs) are sensitive to oxidative damage and they improve the response to oxidative injury and fluidity of the cell membrane [22]. Treatment with EGb 761 could increase the levels of circulating PUFAs in erythrocyte membranes, which enhances the ability to protect against oxidative damage in adult male Wistar rats [23]. Fibrosis in various organs is related to aging. EGb 761 decreased the levels of liver malondialdehyde and metalloproteinase and increased the activity of SOD to prevent the impairment of oxidative stress on aging liver fibrosis [24]. In aged female rats, levels of malondialdehyde and 8-hydroxy-2’-deoxyguanosine (8-OHdG) were found to be decreased with EGb treatment to result in an improvement in cognitive functions [25]. EGb could also decrease malondialdehyde in the rat testes, which is a well-known lipid peroxidation product [21].Other studies [18, 26] have reported similar results to the above in various mouse tissues.

### 1.2 EGb protects DNA from oxidative damage

An excessive level of free radicals can attack the DNA and cause damage, which brings about mutations and consequently, physical manifestations of the damage. EGb 761 (25-100 μg/ml) has been found to improve the capacity of antioxidants in intermittent high glucose by inhibiting ROS generation, 8-OHdG and oxidative DNA damage in human umbilical vein endothelial cells [27]. Rats that have undergone intermittent hypoxia to induce hippocampal DNA damage and treated with EGb 761were reported to have reduced levels of 8-OHdG and have their DNA damage reversed. EGb was found to not only attenuate DNA damage but also stimulate DNA repair [18]. Marques *et al* [28] used a modified comet assay to evaluate DNA repair kinetics in yeast treated with and without EGb. Upon oxidative shock, DNA damage decreased in a dose-dependent manner in experiments of pre-incubation and simultaneous incubation with the extract, indicating a direct protective effect. In addition, the extract improved DNA repair rate following oxidative shock as measured by faster disappearance of comet tails. This suggests that the extract stimulates the DNA repair machinery in its DNA protective action in addition to directly protect DNA from oxidation.

### 1.3 EGb and mitochondrial dysfunction

In 1956, the free radical theory of aging was introduced, which proposed that the accumulation of free radical damage in cells would give rise to aging [29]. Schwarzkopf *et al* [30] reviewed many mechanisms of aging including oxidative damage and mitochondrial dysfunction. For instance, oxidative damage could decrease membrane fluidity, level and function of cardiolipin and acetyl-L-carnitine following the age. In subsequent decades, more and more studies confirm that free radical accumulation relates to aging. Therefore, inhibiting reactive oxygen species could extend life span [31]. Mitochondria are the powerhouse of our cells, which can metabolize O_2_ and glucose to ATP, which provides energy for the cell to function. Mitochondrial dysfunction triggers the release of reactive oxygen species and subsequently induces peroxidative reactions, which lead to mitochondrial biomolecules’ damage. Mitochondrial function is then impaired and could cause neuronal cell death and increased tissue loss, which has also been shown to be associated with aging.

#### The role of EGb in inhibiting mitochondria-induced ROS

Exposing SH-SY5Y cells overexpressing APP to Aβ led to a damage in mitochondrial respiratory capacity and decreased generation of ATP, which consequently triggered cell death signaling pathways [32]. However, treatment of APP cells with EGb for 24 hours resulted in a significant decrease of mitochondria-induced ROS levels [33].

#### The protective role of EGb in mitochondrial respiratory chain

EGb 761 has been shown to stabilize mitochondrial function. Mitochondria isolated from PC12 cells and mice brain cells were used to simulate mitochondrial abnormalities in aging. As low as 0.01 mg/ml EGb 761 could relieve mitochondrial functions. *In vivo*, EGb 761 had a significant protection on mitochondrial respiratory chain complexes I, IV and V only in the 15-month-old mouse group, but had no effect in the 2-month-old mouse group [34]. In PC12 cells, EGb 761 conferred protection on the mitochondrial respiratory complexes I, II, IV and V[35], improved the mitochondrial membrane potential in sodium nitroprusside after an insult and reversed the decrease of ATP production [36]. Other studies [37, 38] reported that EGb 761 prevented the changes of membrane potential and mitochondrial morphology associated with aging in the brain and liver. Intriguingly, Ginkgo B, a constituent of EGb, also had the ability to enhance the activities of complexes I and IV in Aβ25-35 induced oxidative phosphorylation [14], suggesting that Ginkgo B could be the active component mediating mitochondrial protection. Rhein et al [33] compared the mitochondrial complexes I to IV and F1F0 ATP synthase in APP and control cells after pretreatment with EGb for 24h. They found that mitochondrial respiration in APP cells was significantly improved and the level of ATP increase was closely related with the higher activity of complex V by pretreatment with EGb. Furthermore, EGb treatment caused an increased activation of complexes I and III in APP cells. Mitochondrial complex IV activation was down regulated, whereas complex II was not changed after EGb treatment.

#### The role of EGb in inhibiting mitochondria-mediated apoptosis

Shi et al [39] examined the effects of EGb 761 on platelets and hippocampal mitochondria isolated from SMAP8 mice of different age groups. SMAP8 mice were a senescence-accelerated strain of mice. The authors found that EGb 761 prevented the activation of cytochrome c oxidase and the decrease of mitochondrial glutathione and mitochondrial ATP with aging. In platelets, they observed that EGb 761 prevented mitochondrial dysfunction both in the young and old mice. In contrast, EGb 761’s protective effect only could be observed in the old mice. They proposed that this phenomenon may be due to the increased blood brain barrier’s (BBB) permeability with aging. In another study [40], EGb 761 treatment was shown to regulate the expression levels of pBcl-xL and Bax by inhibiting caspase-9 activation to guard against mitochondrial dysfunction in the cochlear of rats.

#### The role of EGb in mitochondrial DNA (mtDNA)

Apurinic/apyrimidinic endonuclease1 (APE1) is a multifunctional enzyme for DNA repair after oxidative stress. Kaur et al found that application of Aβ25-35 caused a decreased level of mitochondrial APE1, but treatment with ginkgo B could reverse this decrease significantly and help the damaged mitochondria repair [14]. EGb was found to enhance the ratio of mitochondrial DNA/nuclear DNA [33], indicating that EGb could up-regulate mitochondrial DNA in APP cells.

#### The role of EGb in mitochondrial membrane potential and intra-mitochondrial Ca2^+^homeostasis

Kwon and colleagues [15] used a model of brain ischemia-reperfusion in gerbils to study how EGb affects Ca^2+^ homeostasis after an ischemic insult. The animals were pretreated with EGb once a day for 28 days before the onset of ischemia. Dihydrorhod-2-AM, an indicator of Ca^2+^, was used to evaluate the level of intramitochondrial Ca^2+^. The authors observed that intramitochondrial Ca^2+^ was increased after ischemia and this elevation was significantly attenuated by EGb 761 (48 mg/kg) pretreatment. Furthermore, using the JC-1 dye to measure mitochondrial membrane potential [41], they reported that EGb 761 (48 mg/kg) could reverse mitochondrial transmembrane potentials reduced by ischemic insult and could mediate neuroprotective effects by inhibiting mitochondrial dysfunction.

**Figure 3.**
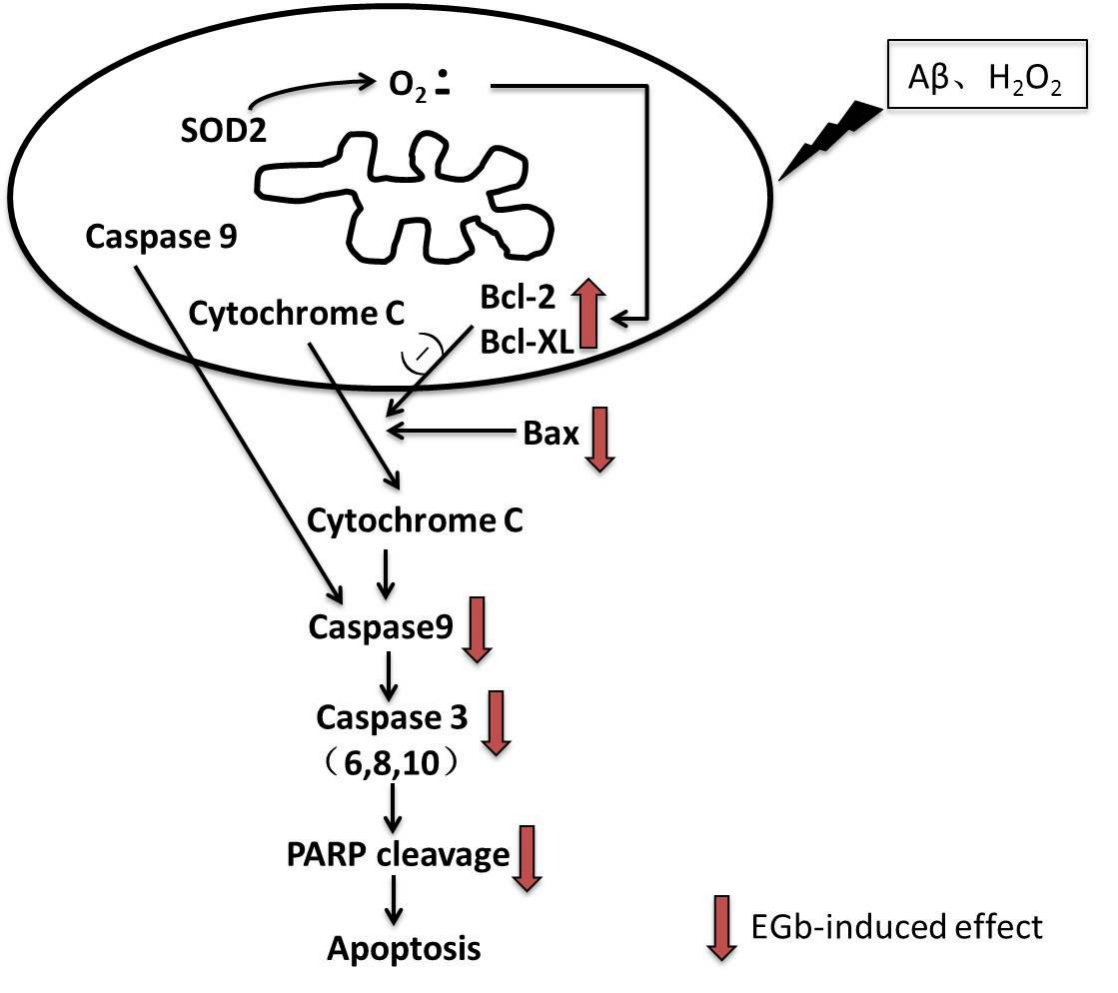
Mitochondria mediated apoptosis Mitochondria are the target of stress injury. The generation of ROS in mitochondria then induces the release of cyt-c by mechanisms related to Bcl-2 family proteins (Bcl-2, Bcl-Xl, Bax, and Bid). Once cyt-c released, it binds to caspase-9 to form a complex which subsequently activates caspase-3 and other caspases, such as caspase-2, -6, -8 and -10. Activated caspase-3 is known to cleave nuclear DNA repair enzymes, which then lead to nuclear DNA damage and finally result in apoptosis. EGb could prevent apoptosis by inhibiting mitochondria-mediated caspases activation.

### 1.4 EGb and apoptosis

#### The protective role of EGb in mitochondia-mediated apoptosis

Increased apoptosis induced by reactive oxygen species (ROS) in the mitochondria plays a key role in aging (Fig. 3). Schindowski et al [43] evaluated whether oxidative stress could change the cell’s susceptibility to apoptosis and whether this change could be inhibited by EGb 761 in old mice. They used ROS to induce apoptotic lymphocytes in the mouse’s spleen and found that EGb 761 (100 mg/kg) treatment for 14 days reduced the number of 2-deoxy-d-Ribose(dRib)-induced apoptotic cells; however, there was no demonstrated benefit for cells in young mice (3months). The benefit was specific to cells from old mice. In an in vitro study, similar results were obtained. Bilobalide is an important element in EGb. Chun and Wu [44] investigated the effect of bilobalideon apoptosis in SH-SY5Y cells. Cells apoptosis was induced with the application of H_2_O_2_, while beta amyloid protein (Aβ) 1-42 and serum deprivation was used to mimic aging-related neurological function impairments. Bilobalide, a biologically active component of EGb, was found to prevent H_2_O_2_-induced cell apoptosis by inhibiting mitochondria-mediated caspases activation; the PI3K/Akt pathway might play an important role in the bilobalide’s protective effects.

#### The protective role of EGb in inhibiting caspases-mediated apoptosis

Aging-related hearing loss most commonly occursin the elderly population. Nevado et al [41] studied the effect of EGb 761 on aging-related cochlear caspase activation in rats. They treated 4-months old and 12-months old rats with EGb 761 for 4 months and 12 months, respectively. They then measured the level of ATP, total superoxide dismutase activity, caspase activities and auditory steady-state responses (ASSR). They found that EGb 761 treatment could improve threshold shifts of ASSR, and this improvement was due to decreased caspase-3/7 activity and caspase-3 gene expression in the rat cochlear. Interestingly, reduced caspase-3/7 activity was seen only in the short-term treatment (4 months) groups but not in the long term (12 months) treated group, indicating that treatment with EGb 761 at a young age is more beneficial than when treatment is administered at middle. Recent study [45] examined the mechanisms of Ginkgolides and Ginkgo flavonoids on cultured rat hippocampal neurons. Flow cytometry and TUNEL staining were used to examine the number of apoptotic neuronal cells. Expression levels of Caspase-3, Caspase-6, Caspase-8, Caspase-9, Caspase-10, Bax and Cytochrome C (Cytc) were also evaluated. Their results showed that pre-treatment with either Ginkgolides or Ginkgo flavonoids could improve cell viability and decrease the levels of Caspase-3, Caspase-6, Caspase-8, Caspase-9, Bcl-2 and Bax. It also can increase the expression of Bcl-2, an inhibitor apoptosis protein. qPCR showed that Ginkgolides and Ginkgo flavonoids pre-treatment could down-regulate Cytc. Furthermore, EGb could influence apoptosis by regulating the level of PARP cleavage and caspase-3 in oral cavity cancer cells [46].

#### The effect of EGb on death receptor mediated apoptosis (FasL/TNF)

**Figure 4.**
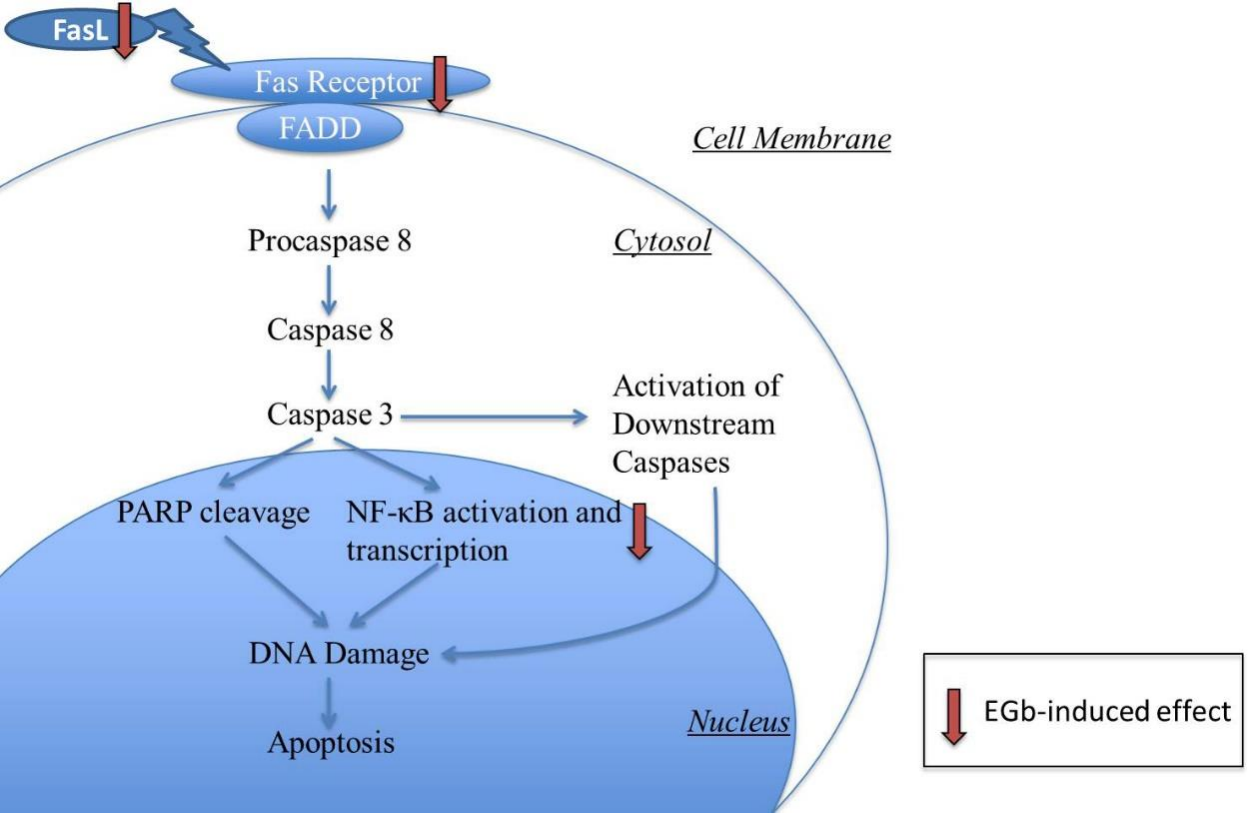
Death receptor mediated apoptosis The extracellular Fas ligand first binds to a receptor, and then binds to FADD protein. This complex activates procaspase-8 into caspase-8. Then, caspase-8 activates caspase-3 and this effector caspase leads to DNA damage and cell death. What is more, Fas ligand such as TNF-α, can also induce NF-κB activation and transcription. EGb protects against apoptosis by decreasing the expression of FasL/FasR and inhibiting NF-κB activation.

FAS Receptor Ligand (FasL) belongs to the genetic family of the tumor necrosis factor (TNF). FasL is rarely expressed in normal cells, but its expression in tumor cells is increased which promotes tumor cells to kill healthy cells. A lot of studies showed that there is a relationship between EGb and death receptor mediated apoptosis (Fig. 4). Jiang XY and colleagues [47] reported that EGb could inhibit apoptosis of pre-cancerous gastric cells by limiting the expression of FasL induced by 100 mg/L N-methyl-N′-nitro-N-nitrosoguanidine (MNNG) solution in elderly rats; this effect was dose-dependent. In another study, the effects of EGb injection on the injury of renal ischemia/reperfusion were observed[48]. The data showed that EGb injection could attenuate the enhanced FasL at 3h after ischemia onset and reduced the apoptosis of renal epithelial tubular cells.

NF-κB has emerged as a major regulator of programmed cell death via apoptosis and necrosis. Its p65 subunits contain the transcriptional activation domain, which can induce the expression of proapoptotic genes such as the death receptor Fas as well as the death-inducing ligands FasL and TNF-α. EGb 761 could inhibit NF-κB activation induced by β-amyloid peptide and then suppresses gene expression of TLR and NF-κB, and reduces the number of apoptotic neuronal cells [49]. Osteoarthritis is another degenerative disorder that commonly affects the elderly. In Chen’s research, EGb761 could prevent cartilage breakdown by inhibiting NF-κB induced death receptor mediated apoptosis in osteoarthritic rat knee [50]. It was demonstrated that EGb attenuated TNFα-induced NF-κB activation in obesity-related metabolic diseases[51].

#### The role of EGb in cell cycle regulation

Tanigawa and colleagues [52] demonstrated that quercetin, a component of EGb, was able to induce p53 phosphorylation, increase expression of p21 and decrease expression of cyclin D1 in HepG2 cells, as well as increase the ratio of Bax/Bcl-2. Their data demonstrated that quercetin inhibited apoptosis through stabilizing p53 and then reactivating the p53-dependent cell cycle in HepG2 cells. A molecular docking study revealed that quercetin, a flavonoid constituent of Ginkgo biloba, was a potent DNA damage inducer in HepG2 cells by interacting with topoisomerase II (Topo II) [53].

EGb has two roles in apoptosis. In normal tissues, it suppresses apoptosis. In cancer cells, it actually induces apoptosisand cell cycle arrest to prolong life span [54]. Treatment of human gastric cancer cells with EGb761, the flow cytometry results showed that gastric cancer cells were accumulated in G0/G1 phase when exposed to EGb761 [55]. Chen et al [56] reported that EGb 761 could inhibit the progression of human colon cancer cells by increasing the number of cells in the G0/G1 phase and reducing cells in the G2/M and S phase. Qian and collegues [57] also reported that EGb conferred an inhibitory effect on the proliferation of gastric carcinoma SGC7901 cells both in vitro and in vivo. The inhibitory effect was dose dependent and possibly depended on inhibiting cell cycle through G1 arrest induction by suppressing cyclin D1 and c-myc expression.

EGB conferred an inhibitory effect on the proliferation of gastric carcinoma SGC7901 cells both in vitro and in vivo. The inhibitory effect was dose dependent and possibly depended on inhibiting cell cycle through G1 arrest induction by suppressing cyclin D1 and c-myc expression.

#### The role of EGb in Ca^2+^ homeostasis

In Dutta-Roy’s research [58], EGb could enhance cAMP levels to regulate intracellular Ca^2+^ concentration and inhibited platelet aggregation in humans.In another study, researchers evaluated the blood viscosity in 48 men aged 60-70 years, and found that EGb could reduce blood viscosity and improve cerebral perfusion in specific areas [59]. Meng and colleagues [60] studied the ability of Ginkgolid B, one of the components of EGb, in PC12 apoptosis. They induced apoptosis by 6-hydroxydopamine (6-OHDA) and examined the concentration change in Ca^2+^ by fluorescent indicator (fluo-3/AM). Their data showed that Ginkolide B could improve apoptosis on PC 12 by decreasing intracellular Ca^2+^ concentrations. Tsai et al [61] reported that EGb decreased the level of lectin-like ox-LDL receptor 1 via inhibiting Ca^2+^, PKC and etc to limitthe oxidized low-density lipoprotein-induced injury in human umbilical vein endothelial cells.

#### The role of EGb in MAPKs signaling pathway

The toll-like receptor 4 (TLR4) play a key role in mediating inflammatory responses in vascular smooth muscles cells during atherogenesis. Lin *et al* [62] reported that phosphorylation of intracellular mitogen-activated protein kinases (MAPKs) was activated by lipopolysaccharide (LPS) and enhanced the of TLR4’s mRNA stability. EGb could reverse this LPS-induced activation of MAPKs phosphorylation and inhibit TLR4 expression to protect human aortic smooth muscle cells. In the liver, EGb could mitigate fibrosis through decreasing p38 MAPK levels to improve hepatocyte apoptosis and liver function [63].

There is a high incidence of diabetes in the elderly, prompting continued research on the disease mechanisms and treatment options. Human aortic endothelial cells (HAECs) were cultured with high glucose for 4 days and then treated the cells with EGb to observe the level of HAECs. They found that high glucose decreased expression of heme oxygenase-1. Meanwhile, EGb could preserve endothelial adhesion by increasing heme oxygenase-1 levels via the p38/MAPK pathway [64]. In another study, Li and his colleagues [65] demonstrated that ginkgolides could increase HIF-1α expression via activating the p42/p44 MAPK pathway in order to protect against hypoxia injury in PC 12 cells. MAPKs signaling pathways also play a key role in cells apoptosis in brain ischemia/reperfusion injury. Jiang *et al* [66] used the middle cerebral artery occlusion rat model and OGD model to examine the effects of bilobalide. Their results showed that bilobalide had the protective abilities by decreasing JNK1/2 levels and p38 MAPK both *in vivo* and *in vitro*. There was another study demonstrating that MAPKs also play a pivotal role in bilateral common carotid occlusion on rats [67].

## 2. The effect of EGb on aging-related diseases

### 2.1 Neuron protection by EGb

Neurodegenerative diseases, like Alzheimer’s disease (AD) and brain ischemia, are significant causes of mortality and morbidity in the elderly, resulting in EGb has been proven to take part in many age-related neurodegenerative diseases.

#### The effect of EGb on cerebrovascular disease

Chronic inflammatory diseases are associated with increases in cardiovascular diseases [68]. Krieglstein and colleagues [69] examined the effects of EGb on local cerebral blood flow and found that EGb could increase blood flow. They thereby speculated that EGb could increase blood flow to protect brain tissue against ischemia or hypoxic damage. Cho *et al* [70] investigated the effect of EGb 761 (100 mg/kg) in middle cerebral artery occlusion (MCAO) rat models. They collected rat brains and analyzed the infarct volumes and potential mechanisms after 24 hours of reperfusion and found that EGb 761 could significantly decrease infarct volume and decrease the protein level of pAkt, as well as its downstream targets such as pBad and pFKHR. EGb 761 was also found to activate caspase-3. In another ischemic rat model induced by four-vessel occlusion [71], EGb 761 treatment could decrease the level of lipid peroxidation. As a huge influx of calcium into the cell activates downstream pathways of ischemia-reperfusion injury, Koh *et al* [72] found that EGb 761 could prevent the decrease in the level of hippocalcin, a sensor of neuronal intracellular calcium concentration, to confer neuroprotection. Parvalbumin, another calcium buffering protein, was reported to decrease after ischemia, but EGb 761 was found to reverse this effect [73].

Traditional experimental methods such as behavioral test and 2, 3, 5-triphenylte trazolium chloride (TTC) staining for evaluating ischemic outcome, although useful, have their limitations. With the rapid development of Magnetic Resonance Imaging (MRI), more studies are beginning to include the use of this new technology. Zhang and colleagues [74] used magnetic resonance diffusion-weighted imaging (DWI) to evaluate ischemic injury and found that EGb could enhance the value of apparent diffusion coefficient and average diffusion coefficient in the central and peripheral zones. Additional studies found that the levels of cAMP response element binding protein and Akt phosphorylation were increased in the ischemic brain after EGb treatment. Moreover, brain-derived neurotrophic factor (BDNF) levels were increased. They thereby concluded that EGb could activate the pathway Akt-Creb-BDNF to mediate its protective role. In another study [30], after injecting bilobalide into the striatum through microdialysis, brain infarct volume was decreased significantly. Bilobalide, a component of EGb, was found to protect the brain by decreasing the level of extracellular glutamate. Moreover, bilobalide, given through the intraperitoneal route at one hour before MCAO onset, could prevent the loss of ATP and was beneficial to the brain. Bilobalide’s ability to suppress ischemia-induced glutamate release was also reported in another *in vivo* study [75]. In 2005, Zeng *et al* [76] assessed 10 clinical trials (about 792 patients) and found that there was no convincing evidence to support EGb’s promotion of stroke recovery; three trials reported adverse results. However, EGb’s neuroprotective effect should not be dismissed altogether due to the small number trials conducted and patients enrolled per study.

#### The effect of EGb on Alzheimer’s disease

AD is an age-related neurodegenerative disease that results in cognitive impairment [77]. The hippocampus is thought to play an important role in cognition and is the center for memory processing and synaptic plasticity. For instance, in Mix’s research[78], patients who took 180 mg/day of EGb 761 for 6 weeks showed a significant improvement on tasks assessing processing speed. In fact, EGb is widely used in the treatment of Alzheimer’s disease [79]. Williams and colleagues [80] observed that EGb 761 could increase the neuronal excitability, and efficacy of synaptic plasticity in the hippocampus of aged rats, but not in young rats. It is widely accepted that β-amyloid precursor protein and amyloid β-peptide play key roles in the development of AD. Eckert *et al* [38] reported that EGb 761 decreased the level of β-amyloid precursor protein in PC12 cells, which bore an Alzheimer’s disorder-related mutation. Using a two-month-old APP/PS1 transgenic mouse model of AD [81], Wan *et al* supplemented EGb 761 daily for six months and detected levels of Aβ and inflammatory cytokines thereafter. They reported a reduction in the levels of insoluble Aβ and pro-inflammatory inducible nitric oxide synthase while arginase-1 was increased. These results were supported by treating BV2 microglial cells with EGb 761. Taken together, their study demonstrated that EGb 761 played a protective role in APP/PS1 mice by regulating the expression of Aβ and inflammatory cytokines. EGb 761 was also able to suppress the toxic effects induced by Aβ peptides in the hippocampal cells of the aging rat by inhibiting the activation of protein kinase C (PKC), which blocks stimulated sodium nitroprusside (SNP) [82]. Yao and colleagues [83] found that free cholesterol might be involved in the production of β-amyloid precursor protein and the amyloid β-peptide. They demonstrated that EGb 761 could decrease the free cholesterol in rats and suppress the expressions of the β-amyloid precursor protein and amyloid β-peptide *in vivo* and *in vitro*.

In Stein C’s research [84], they found that EGb 761(100 mg/kg/day,) could reduce choline levels and play a neuroprotective role in aged rats by oral gavage for 14 days. In another study, researchers reported that EGb 761 could increase BDNF levels and improve cognitive functions in aging female rats [25].

In addition to basic research, there have been many clinical trials on EGb. In Cieza A’s clinical trial [85], they subjected 66 healthy volunteers aged 50 to 65 years to EGb 761 treatment or placebo for 4 weeks. They found that EGb 761 could significantly improve patients’ self-reported mental health and quality of life. There was a 6-month long clinical trial that demonstrated EGb 761(180 mg/day) could improve neuropsychological, memory and cognitive processes [86]. Kaschelet al [87] examined 188 middle-aged healthy volunteers’ memories after taking Egb for weeks. He found that patients’ quantity recall and qualitative recall were both significantly improved with EGb 761 treatment, but there was no difference in everyday memory performance, such as that of a driving route, between the treatment and placebo group. This research demonstrated that EGb761 could improve free recall in middle-aged people. Kaoru *et al* [88] found that EGb could improve middle-aged women’s working memory performance by increasing cerebral blood oxygenation to the prefrontal cortex. In the last year, Tan MS and colleagues [89] studied the effects of EGb 761 in 2561 patients diagnosed with AD, dementia or mixed dementia based on internationally diagnostic criteria. They performed a meta-analysis and found that EGb 761 (240 mg/day) treatment for 22-26 weeks was observed to improve cognitive impairment and dementia. Similar reports [90, 91] have also found EGb 761 to be beneficial for those enrolled in the study. However, a French clinical study by Vellas Bruno et al showed otherwise [92]. They enrolled 2854 participants in total who were over 70 years old with reported memory complaints to their doctors occurring spontaneously between 2002 and 2004. These patients were supplied standardized EGb 761 (120 mg, twice a day) and followed-up for five years. The authors observed that EGb 761 could not decrease the risk of progression to AD after long-term use. A similar conclusion was reached by Persson and colleagues [93] who followed 40 healthy adult volunteers with a mean of 68 years old for two years. The supplied EGb could not provide a quantifiable beneficial effect on their memory performance. In 2007, Canter and Ernst [94] reviewed 15 randomized clinical trials and found that only 1 out of 5 acute studies and 1 of 6 longer-term studies reported beneficial effects, suggesting that there was no convincing evidence to conclude that EGb had any beneficial effects on cognitive function for those under 60 years old.

#### The effect of EGb in Parkinson’s disease

Parkinson’s disease (PD) is a common aging related disease resulting in the progressive loss of dopamine neurons. In Kim’s research [95], EGb 761 pretreatment could decrease dopamine neuron loss in the substantia nigra and improve the behavioral deficit in a rat Parkinson’s disease model. Kang and colleagues [96] reported that EGb 761 maintenance kept the stability of mitochondrial membrane potential and decreased caspase-3 levels in paraquat-induced apoptosis of PC12 cells; it was an experimental basis for using EGb 761 to treat PD. In another animal models of PD, which was induced by 1-methyl-4-phenylpyridinium, the authors found that EGb 761 could protect against neurotoxicity via preventing changes of copper content in the striatum, midbrain and hippocampus [97]. Tanaka systematically reviewed the effect of EGb in animal models of PD in 32 English language articles and found that most studies reported focused on EGb protection against neurotoxin, anti-oxidative stress, anti-apoptotic and reduced loss of the neurotransmitter, dopamine, in the striatum [98].

### 2.2 The effect of EGb on anti-cancer

Cancer has a huge impact all over the world. According to the NIH, an estimated 1.7 million new cases of cancer will be diagnosed in the United States and 0.6 million people died from cancer in 2016. Therefore, new therapeutics for the treatment of cancer is critical. Several studies have focused on the anti-apoptotic effect of EGb. Ahmed and colleagues [5] examined the efficacy of EGb in the suppression of hepatocellular carcinoma (HCC) in rats. EGb was found to improve the histological features of the liver tissue significantly. Gene expressions of ING-3 were also up-regulated while Foxp-1 was down-regulated in the liver after EGb treatment. Further, EGb could decrease the levels of alpha-fetoprotein (AFP), glypican-3 (GPC-3) and carcinoembryonic antigen (CEA) in HCC rats, suggesting that the anti-cancer efficacy of EGb was induced through its anti-proliferative and apoptotic properties in the HCC animal model.

Han et al [99] studied the activity of Ginkgo bilobaexocarp extracts (GBEE, the exocarp of Ginkgo nuts) against Lewis lung cancer (LLC). They first used the MTT method to detect the number of LLC cell proliferation after treatment with GBEE. Their results showed that GBEE could suppress the growth of LLC cells by regulating the expressions of Wnt3a and β-catenin *in vitro* in a dose dependent manner. Further, using C57BL/6J mice, they evaluated the expression levels of certain proteins such as VEGF, Wnt3a and p-Akt/Akt and obtained similar results. GBEE could therefore suppress metastasis of lung tumor since the protein expressions of β-catenin, p-AKT/AKT VEGF, VEGFR were decreased. Correspondingly, the mRNA levels of VEGF and VEGFR2 were decreased also, indicating that GBEE plays an anti-tumor role through inhibition of tumor angiogenesis. The mechanisms of those effects were associated with blocking the Wnt/β-catenin-VEGF signaling pathway in LLC. A clinical trial with regards to the safety and efficacy of EGb with sorafenib in patients with advanced HCC by Cai and colleagues [100] found that the maximum combination of 240 mg EGb (QD) and 400 mg sorafenib (BID) was safe and tolerable for HCC patients.

Qian Y and colleagues [101] studied the effect of EGb in human gastric carcinoma SGC7901 cells *in vitro* and in nude mice. They reported that there was a dose and time dependent relationship of EGb in inhibiting the proliferation of gastric carcinoma SGC7901 cells. Using flow cytometry, they found that EGb increased the percentage of cells in the G1 stage, but decreased those in the S stage. Moreover, the level of mRNA and protein cyclin D1 and c-myc genes were down-regulated significantly in EGb-treated cells, suggesting that EGb could inhibit the cell cycle progression by down-regulating cyclin D1 and c-myc expressions. Liu and colleagues [102] reported similar results when they applied EGb 761 in gastric cancer cells. However, the underlying mechanisms of EGb’s effects were restricted to the KSR1-mediated ERK1/2 pathway.

EGb is also involved in several other cancers including breast cancer, non-small cell lung cancer (NSCLC), colon cancer and prostate cancer. Zhao et al [39] reported that GEb treatment could increase the expression of cytochrome P450(CYP)1B1 in MDA-MB-231 human breast cancer cells. Park *et al* [103] also found that EGb mediated cytotoxic effects in the breast cancer cell line MDA-MB-231 by activating caspase-3 and altering the mRNA levels of apoptosis-related genes Bcl-2 and Bax. Tsai and colleagues [104] analyzed the effect of EGb 761 in NSCLC patients. They found that EGb 761 could prolong patient survival by suppressing Heat-shock proteins 27. EGb could also decrease the migration ability of cancer cells by activating the AKT and p38 MAPK pathways. EGb 761 could suppress the growth of human colon cancer cell line HT-29 in a dose and time dependent manner [105]. EGb 761 (80 and 320 mg/L) could elevate the number of cells in the G0/G1 phase but decreased them in the G2/M and S phases, which could be attributed to the activation of protein caspase-3, increase in p53 mRNA expression and decreased bcl-2 mRNA expression. With regards to prostate cancer cells, EGb has been found to induce apoptosis as well as inhibit cancer survival genes such as Bcl-2, Bcl-xL, survivin and Cyclin D1 [53]. Park *et al* examined some death-associated protein and found that some flavonoids of EGb could induce cell apoptosis in melanoma cells via increasing the levels of caspase-3, caspase-9 and p53. Hence, EGb can be a potent anticancer therapeutic through its actions such as the induction of apoptosis, regulation of cell cycle progression and gene expression.

### 2.3 The effect of EGb on the cardiovascular system

Ischemic heart disease is the top disease “killer” during 2000 to 2012 according to the WHO. Kubota and colleagues [106] examined the effect of EGb on the level of intracellular calcium in rat thoracic aorta endothelial cells and found that EGb could enhance the level of Ca^2+^ in those cells to produce vasodilation. Liuet al [4]made use of D-galactose to induce an aging phenotype in cardiomyocytes. They reported that treatment with EGb 761 could decrease the number of SA-β-gal positive cells as well as decrease diastolic Ca^2+^ and increase its reuptake. They also observed that SERCA2a played an important role in improving the diastolic dysfunction of aging rats. In Kubota’s study [107] spontaneously hypertensive rats (SHR) were fed with a diet that contained 0.05%-0.5% ginkgo for a period of 30 days. The authors noticed that the level of intracellular calcium was enhanced in endothelial cells, which seemed to improve the impairment of dilatory function in these cells. Han and Li [108] reported that EGb could inhibit angiotensin II and hypoxia induced vascular endothelial cells damage by decreasing the level of Ca^2+^ and mitochondrial membrane potential. In 2000, Campos-Toimil *et al* [109] examined the effect of EGb 761 in vascular endothelial cells and demonstrated that EGb could restrain the activity of type 4 phosphodiesterase by decreasing agonist-induced enhancement of Ca^2+^, suggesting that Ca^2+^ signaling plays a key role in mediating the normal function of human endothelial cells. In a clinical trial by Rodriguez *et al* [110], the levels of free radical scavenging enzymes and risk factors were examined in eight patients who had undergone an aortocoronary bypass and were treated with EGb for two months. The authors reported that EGb could increase the activation of superoxide dismutase and decrease the levels of oxLDL/LDL thereby reducing atherosclerotic nanoplaque formation. A similar clinical trial by another group yielded comparable results [111]. Brinkley and colleagues [112] examined the effect of EGb on blood pressure in more than 3000 elderly participants wherein half of those enrolled were hypertensive. Their results however, indicated that EGb did not decrease the blood pressure in both elderly men and women.

## Conclusion

EGb is a highly complex compound that consists of several components, which requires systematically analyzing each component’s effects to obtain the best possible combination of the compound for health benefits and improving disease symptoms.

Based on the reviewed information regarding EGb’s effects *in vitro* and *in vivo*, most have reported very positive outcomes with strong statistical analyses, indicating that EGb must have some sort of beneficial effect. However, information from the reported clinical trials involving EGb are hardly conclusive since many do not include information such as the participant’s age and physical condition, drug doses administered, duration of drug administered as well as suitable control groups for comparison. We therefore call on clinicians and clinician-scientists to establish a set of standard and reliable standard operating procedure for future clinical studies to properly evaluate EGb’s effects in the healthy and diseased person since it is highly possible it possesses beneficial effects.
